# Re: Neal D. Shore, Joseph Renzulli, Neil E. Fleshner, et al. Active Surveillance plus Enzalutamide Monotherapy vs Active Surveillance Alone in Patients with Low-risk or Intermediate-risk Localized Prostate Cancer: The ENACT Randomized Clinical Trial. JAMA Oncol 2022;8:1128–36: *Let’s Not Go Back in Time! What Have We Learned in the Treatment of Localized Prostate Cancer? Applicability of the ENACT Randomized Clinical Trial in Clinical Practice*

**DOI:** 10.1016/j.euros.2022.09.022

**Published:** 2022-11-14

**Authors:** Juan Gómez Rivas, Giorgio Gandaglia, Francesco Montorsi

**Affiliations:** a Department of Urology, Hospital Clinico San Carlos, Madrid, Spain; b Young Academic Urologists, European Association of Urology, Arnhem, The Netherlands; c Unit of Urology, Division of Oncology, IRCCS San Raffaele Hospital, Vita-Salute San Raffaele University, Milan, Italy

Androgen manipulation in patients with prostate cancer (PCa) was first proposed by Huggins and Hodges in 1941 [1], when their pioneering investigations demonstrated that PCa is an androgen-sensitive disease and that administration of testosterone increased prostate growth in animal models. Over the years, the role of androgen deprivation therapy (ADT) emerged in the setting of recurrent and/or metastatic disease or as an adjuvant or neoadjuvant option in men receiving curative-intent therapies. Currently, international clinical guidelines do not recommend starting any form of androgen manipulation in men with de novo localized PCa who are not candidates for definitive treatments such as radiotherapy [2]. Conversely, active surveillance (AS) has become popular for reducing the risk of side effects associated with radical prostatectomy or radiotherapy without losing the so-called window of curability, and is now recommended by virtually all clinical guidelines for men with indolent disease. The safety of AS is supported by large randomized controlled trials (RCTs). For example, the Prostate Testing for Cancer and Treatment (ProtecT) trial [3] randomized1643 patients with localized PCa to active monitoring (AM; an intermediate pathway between AS and watchful waiting), radical prostatectomy, or radiation therapy. The results show that death from PCa was as low as 1% at a median of 10 yr of follow-up, irrespective of the treatment assigned. Of note, AM was generally well accepted (88% compliance) and approximately 44% of the patients had not received radical treatment and avoided adverse effects (AEs) at 10 yr after diagnosis. The European Organisation for Research and Treatment of Cancer (EORTC) 30891 trial [4] compared deferred ADT versus immediate ADT in 985 patients with T0–4 N0–2 M0 disease and failed to find significant differences in cancer-specific survival owing to the low risk of cancer-specific mortality in this setting. If the premises are that (1) low-risk disease is not associated with a risk of death, (2) delayed therapeutic pathways are well tolerated and offer good quality of life (QoL), and (3) ADT does not improve disease-specific survival in this setting, why should we consider novel androgen receptor-targeting agents (ARTAs) in patients suitable for AS?

ENACT, a recently published phase 2, open-label RCT [5], evaluated a large cohort of patients with low- or intermediate-risk PCa and assessed the efficacy and safety of enzalutamide monotherapy plus AS versus AS alone. The trial demonstrated that despite the substantial risk of side effects, enzalutamide was associated with a significant treatment response, defined as pathological or therapeutic disease progression at 2-yr follow-up. Although this represents one randomized trial demonstrating the efficacy of hormonal manipulation in AS-eligible patients, the question to ask is how these findings would impact everyday clinical practice. Supporters of this study might argue that the use of an oral systemic treatment that might be tolerated would improve compliance with AS and reduce the number of patients seeking curative-intent therapies because of anxiety. However, some points should be considered.

First, although the authors define their experimental arm as “AS plus enzalutamide”, this wording does not reflect the reality of the facts. Patients included in the experimental arm were actively treated with enzalutamide for 1 yr and were not just surveilled. Applying the same criterion, one might state that patients with low-risk PCa receiving high-intensity focal ultrasound are treated with “AS plus focal therapy”. The use of surveillance concepts and follow-up protocols does not justify the concomitant administration of toxic treatments that would eventually have an impact on short-term pathologic or therapeutic endpoints without improving survival.

Second, what about the side effects of a systemic treatment? Are these worth the value? Historically, ADT represented the most common cause of severe male hypogonadism, with a negative impact on lipid, glucose, muscle, and bone metabolism, resulting in a large variety of AEs, including obesity, metabolic syndrome, osteoporosis, sarcopenia, diabetes mellitus, cardiovascular disease, gynecomastia, and sexual dysfunction. Several studies showed that ADT after only 6 mo was associated with higher risk of diabetes mellitus, cardiovascular disease, and myocardial infarction. Loss of muscle mass might result in a decrease in muscle strength, an increase in fragility, a decline in functional performance, and loss of independence. Bone mass density declines within months of ADT initiation, with a maximum decrease of 5–10% within the first year [6], [7], [8], [9], [10]. In the ENACT RCT [5] enzalutamide was administered without concomitant ADT for a 1-yr period. Of note, this represents a unique opportunity to assess the side effects of ARTAs alone. Although the authors of the trial concluded that enzalutamide is well tolerated, they found fatigue in more than half of the patients, and gynecomastia and other related symptoms in one-third. It is also worth noting that in this population with low- and intermediate-risk disease, erectile dysfunction was reported by up to 18% of patients. This compares unfavorably when considering men treated with focal therapy or nerve-sparing approaches, which represent curative-intent therapies delivered directly to the prostate rather than toxic systemic treatments.

Finally, the cost of oral enzalutamide 40 mg is approximately €13 000 for 120 capsules, which represents an annual cost of more than $100 000; an indirect comparison to prostatectomy or radiotherapy (from €18 000 to €20 000), taking into consideration that ADT is not a curative treatment, means that the ENACT strategy does not make sense from a cost-effectiveness point of view.

According to the final ENACT statement, “Enzalutamide may provide an alternative treatment option for patients undergoing AS”. However, according to the issues we have highlighted and without strong data for a clinical benefit such as metastasis-free survival, overall survival, or QoL, enzalutamide cannot be an option for these patients.

In conclusion, we should not go back in time and treat localized PCa with any form of androgen manipulation when level 1 evidence supports the safety of AS or AM and of local therapies in this setting. Offering a treatment with a high individual cost in terms of toxicity and AEs and a substantial economic burden on health care systems without an impact on survival or QoL just makes no sense in an era when widespread dissemination of multiparametric resonance imaging, novel markers, and minimally invasive treatments allows us to offer tailored treatment selection and ultimately improve our patient care.

  ***Conflicts of interest***: The authors have nothing to disclose.
